# Efficacy and Safety of Duodenal Stenting for Malignant Gastric Outlet Obstruction: Insights From a 15‐year Single‐Center Experience

**DOI:** 10.1002/deo2.70192

**Published:** 2025-08-25

**Authors:** Masatoshi Murakami, Nao Fujimori, Akihiko Suenaga, Takahiro Ueda, Shotaro Kakehashi, Akito Furuta, Akihisa Ohno, Kazuhide Matsumoto, Yu Takamatsu, Keijiro Ueda, Yoshihiro Ogawa

**Affiliations:** ^1^ Department of Medicine and Bioregulatory Science Graduate School of Medical Sciences, Kyushu University Fukuoka Japan; ^2^ Department of Gastroenterology Clinical Research Institute, National Hospital Organization Kyushu Medical Center Fukuoka Japan

**Keywords:** complication, duodenal stent, malignant gastric outlet obstruction, neutrophil‐to‐lymphocyte ratio, stent patency

## Abstract

**Objectives:**

Endoscopic duodenal stent (DS) placement has become a primary palliative approach for malignant gastric outlet obstruction (MGOO), offering minimally invasive symptom relief. However, complications and risk factors for stent dysfunction and prognostic indicators of survival are not fully elucidated.

**Methods:**

We retrospectively analyzed 114 patients who underwent initial DS placement for MGOO at Kyushu University Hospital between January 2010 and October 2024. Clinical outcomes, stent patency, and survival predictors were analyzed. The primary endpoint was stent patency; overall survival (OS) was a secondary endpoint.

**Results:**

Pancreatic cancer was the most common underlying malignancy (77.2%). Technical and clinical success rates were 99.1% and 84.1%, respectively; the overall clinical success rate reached 91.2% after additional stenting in initially unsuccessful cases. Adverse events occurred in 12.3% of patients. Among 104 patients with overall clinical success, 18 patients (17.3%) experienced stent dysfunction. Median stent patency and OS were 14.8 and 2.8 months, respectively. Pre‐existing biliary stricture and stent placement across the pylorus were significantly associated with reduced stent patency. High neutrophil‐to‐lymphocyte ratio predicted poorer survival, whereas type III stenosis and post‐stenting chemotherapy were linked to improved survival. No significant differences in outcomes were observed between early and late treatment periods. Reintervention with DS was safe and effective, and 93.3% of patients maintained GOO control with DS alone.

**Conclusions:**

Duodenal stenting is a safe and effective palliative intervention for MGOO. Incorporating inflammation‐based biomarkers and individualized treatment strategies can help optimize patient selection and improve survival outcomes.

## 1 Introduction

Malignant gastric outlet obstruction (MGOO) is a common complication in advanced gastrointestinal malignancies. While traditionally managed with surgical gastrojejunostomy (GJ), endoscopic placement of self‐expandable metal stents (SEMS) has emerged as a preferred palliative strategy due to its minimally invasive nature, faster symptom relief, and shorter recovery time, making it particularly suitable for patients with limited life expectancy. Several recent studies, including multicenter analyses, have validated the efficacy of duodenal stent (DS) placement, reporting technical success rates exceeding 90% and clinical success rates ranging from 80% to 90% [1, 2].

Despite these favorable outcomes, stent dysfunction remains a significant concern, with reintervention rates of 20%–44% [3]. Procedure‐related adverse events (AEs) and stent dysfunction, such as tumor ingrowth, migration, obstruction, and food impaction, are particularly problematic in patients with prolonged survival due to advances in chemotherapy. Among the contributing factors, several studies highlighted the anatomical location of the obstruction site as an important determinant of stent dysfunction and overall clinical outcomes [4, 5].

Beyond anatomical and technical considerations, systemic biomarkers have garnered growing interest for their prognostic utility and potential to inform personalized treatment approaches [6]. Among these biomarkers, the neutrophil‐to‐lymphocyte ratio (NLR) and the Glasgow Prognostic Score (GPS) are widely used due to their accessibility and cost‐effectiveness. Both have been associated with poor prognosis in various cancers, including in patients undergoing palliative interventions for MGOO [4, 7]. In addition to NLR, other inflammation‐based markers, such as C‐reactive protein/albumin ratio (CAR), platelet‐to‐lymphocyte ratio (PLR), and lymphocyte‐to‐monocyte ratio (LMR), have shown prognostic significance in various malignancies [8, 9, 10, 11], although their roles in MGOO remain unclear.

In light of the increasing emphasis on integrating anatomical, technical, and systemic considerations into clinical decision‐making, we conducted a retrospective study to assess long‐term outcomes following DS placement for MGOO at a single high‐volume tertiary center over a 15‐year period. We aimed to identify predictors of stent patency and overall survival (OS), with a specific emphasis on inflammation‐based biomarkers, treatment era, and the impact of post‐stenting chemotherapy.

## 2 Methods

### 2.1 Data Collection and Definitions

**FIGURE 1:**
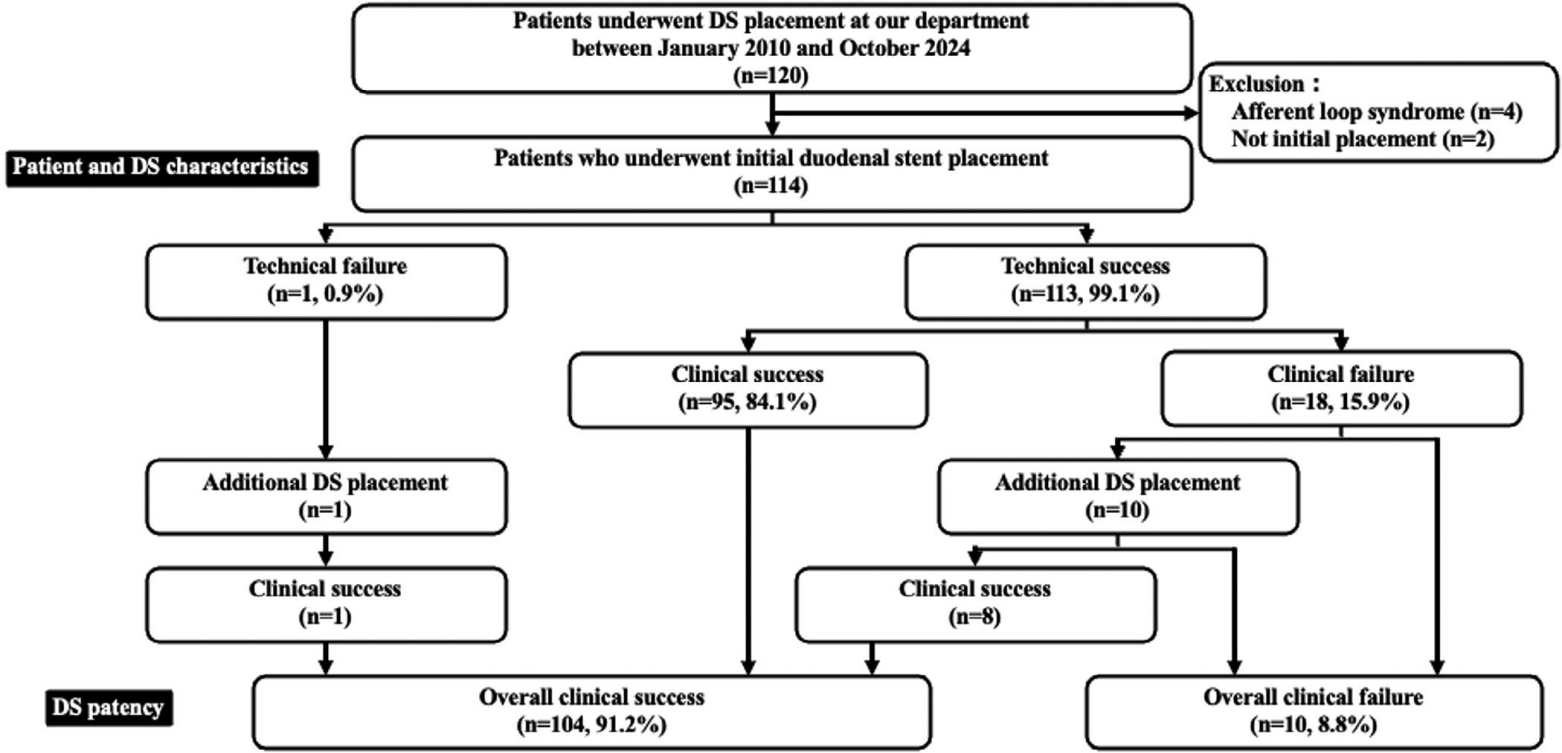
Flow diagram of patient selection and treatment algorithms in the present study. DS, duodenal stent.

A retrospective analysis was conducted on 120 consecutive patients who underwent DS placement for MGOO at Kyushu University Hospital between January 2010 and October 2024. After excluding six cases—four with afferent loop syndrome and two without initial DS placement—114 patients were included (Figure 1).

The primary endpoint was stent patency; OS was secondary. GOO severity was assessed using the Gastric Outlet Obstruction Scoring System (GOOSS) [12], and clinical success was defined as achieving a GOOSS score of 3 after stenting. Stent dysfunction was defined as the recurrence of obstructive symptoms attributable to stent‐related complications. Stent patency was defined as the duration from initial DS placement to symptom recurrence, last follow‐up, or death. In Kaplan–Meier analysis, deaths and follow‐up discontinuations without documented dysfunction were censored. Only clinically documented stent dysfunctions were considered events, focusing on device‐specific durability over general prognosis.

Duodenal stenosis was classified anatomically by its relation to the ampulla of Vater: Type I (proximal to the ampulla), Type II (involving the second part, including the ampulla), and Type III (distal, sparing the ampulla) [13]. Stent selection was based on anatomical features, with various commercially available DSs used at the endoscopist's discretion (details in Supporting Information). Inflammation‐based markers, including NLR, CAR, PLR, and LMR, were calculated and dichotomized based on median values (formulas provided in Supporting Information). For era‐based analysis, the early period was defined as January 2010 to December 2017, and the late period as January 2018 to April 2024.

This study was approved by the Institutional Review Board of Kyushu University Hospital (23345–00). In line with the Ethical Guidelines for Medical and Health Research Involving Human Subjects in Japan, study information—including objectives, data use, and opt‐out procedures—was disclosed on the hospital website (https://www.intmed3.med.kyushu‐u.ac.jp/), allowing patients to decline participation.

### 2.2 Statistical Analysis

All statistical analyses were performed using the R software (v4.3.1; http://r‐project.org). Kaplan–Meier plots with log‐rank testing were used to analyze DS patency and OS. Univariate and multivariate analyses using Cox proportional hazards regression models were used to identify prognostic factors. For categorical variables such as type of stenosis, we created dummy variables and conducted separate multivariate Cox models, each comparing one stenosis type against the combination of the other two.

Variables with *p* < 0.05 on the univariate analysis were further included in the multivariate analysis. Statistical significance was set at *p* < 0.05. To adjust for confounding in evaluating post‐stenting chemotherapy, we conducted 1:1 propensity score matching (PSM) using key clinical covariates. Matching procedures and balance assessments are detailed in Supporting Information.

## 3 Results

### 3.1 Patient and Stent Characteristics

Baseline characteristics of the 114 patients are summarized in Table 1. The most common underlying disease was pancreatic cancer in 88 patients (77.9%), followed by biliary tract cancer in 11 patients (9.6%) and duodenal cancer in 6 patients (5.3%). Most patients had normal anatomical configurations; however, three patients (2.6%) had undergone Billroth I and one (0.9%) had undergone Billroth II reconstruction.

**TABLE 1:** Characteristics of patients and initial duodenal stent (DS).

Factor	*n* = 114
Age at DS placement (years), median [range]	70.0 [42.8–86.8]
Sex Men/Women	83 (72.8)/31 (27.2)
ECOG performance status	
PS 0	14 (12.3)
PS 1	53 (46.5)
PS 2	29 (25.4)
PS 3‐4	18 (15.8)
Primary site	
Pancreas	88 (77.9)
Biliary tract	11 (9.6)
Duodenum	6 (5.3)
Other	9 (7.9)
Gastric tract	
Normal	110 (96.5%)
Altered anatomy	4 (3.5)
Stenosis site	
Type I	41 (36.0)
Type II	35 (30.7)
Type III	38 (33.3)
Ascites	
None	42 (36.8)
Mild	46 (40.4)
Moderate–Severe	26 (22.8)
Dissemination	
Absence	71 (62.3)
Presence	43 (37.7)
Presence of biliary stricture	
None	25 (21.9)
Before duodenal stenosis	51 (44.7)
Simultaneously with duodenal stenosis	29 (25.4)
After duodenal stenosis	9 (7.9)
DS type	
Uncovered type	112 (98.2)
Covered type	2 (1.8)
DS length	
6 cm	19 (16.7)
8 cm	25 (21.9)
9 cm	9 (7.9)
10 cm	23 (20.2)
12 cm	28 (24.6)
15 cm	10 (8.8)
Site of DS placement	
Across the pyloric ring	44 (38.6)
Duodenum	70 (61.4)
Anti‐tumor treatment before DS	
Not administered	38 (33.3)
Administered	76 (66.7)
Anti‐tumor treatment after DS	
Not administered	61 (53.5)
Administered	53 (46.5)
Era	
2010–2017	34 (29.8)
2018–2024	80 (70.2)
GPS	
GPS 0–1	53 (46.5)
GPS 2	61 (53.5)
NLR, median [range]	5.2 [0.9–30.1]
CAR, median [range]	0.7 [0–8.4]
PLR, median [range]	1.6 [0.1–10.3]
LMR, median [range]	2.1 [0.5–20.7]

Abbreviations: CAR, C‐reactive protein‐to‐albumin ratio; DS, duodenal stent; GPS, Glasgow prognostic score; LMR, lymphocyte‐to‐monocyte ratio; NLR, neutrophil‐to‐lymphocyte ratio; PLR, platelet‐to‐lymphocyte ratio.

In 44.7% of cases, biliary stricture preceded duodenal obstruction. A DS longer than 10 cm was placed in 56% of patients, and in 40% of patients, the stent extended beyond the pyloric ring. The majority of stents used were the uncovered type (98.2%). Regarding device selection, 91 patients received a Niti‐S DS, 18 received a WallFlex DS, four received an Evolution DS, and one patient received a JENTLLY NEO DS. In five patients, two stents were inserted during the same session.

### 3.2 Clinical Outcomes

Technical success was achieved in 113 patients (99.1%), and initial clinical success was observed in 95 patients (84.1%) (Table 2). Of the 18 clinical failures, 11 patients (including 1 technical failure) underwent additional stenting during the same admission, resulting in clinical success in nine of those cases. Clinical failure was attributed to stent shortening (*n* = 4), dilation failure (*n* = 2), kinking (*n* = 2), migration (*n* = 1), and perforation (*n* = 1). Consequently, the overall clinical success rate, including these cases, reached 91.2% (104/114) (Figure 1). The mean GOOSS improved from 0.96 ± 1.12 before the procedure to 2.84 ± 0.56 post‐procedure. The median time to oral intake after the procedure was 2 days (range: 1–12). Although formal quality‐of‐life (QOL) metrics were unavailable, functional status was assessed using GOOSS. Importantly, 93.3% (97/104) of patients with overall clinical success maintained oral intake until death or study endpoint.

**TABLE 2:** Clinical outcomes of duodenal stent (DS).

	*n* = 114
Time to oral intake (days), median [range]	2 [1–12]
Technical success	113 (99.1)
Clinical success	96 (84.2)
Overall clinical success	104 (91.2)
GOOSS score before DS placement, mean ± SD	0.96 ± 1.12
GOOSS score after DS placement, mean ± SD	2.84 ± 0.56
Procedure‐related adverse events	14 (12.3)
Obstructive jaundice/ cholangitis	7 (6.1)
Obstructive pancreatitis	3 (2.6)
Bleeding	3 (2.6)
Perforation	1 (0.9)
Mallory‐Weiss syndrome	1 (0.9)
Stress cardiomyopathy	1 (0.9)
Patients with overall clinical success	n = 104
Stent dysfunction	24 (23.1)
Tumor ingrowth	14 (13.5)
Stent shortening	4 (3.8)
Poor stent expansion	2 (1.9)
Stent kinking	2 (1.9)
Stent migration	1 (1.0)
Increased stricture length	1 (1.0)

Abbreviations: DS, duodenal stent; GOOSS, gastric outlet obstruction scoring system; SD, standard deviation.

**FIGURE 2:**
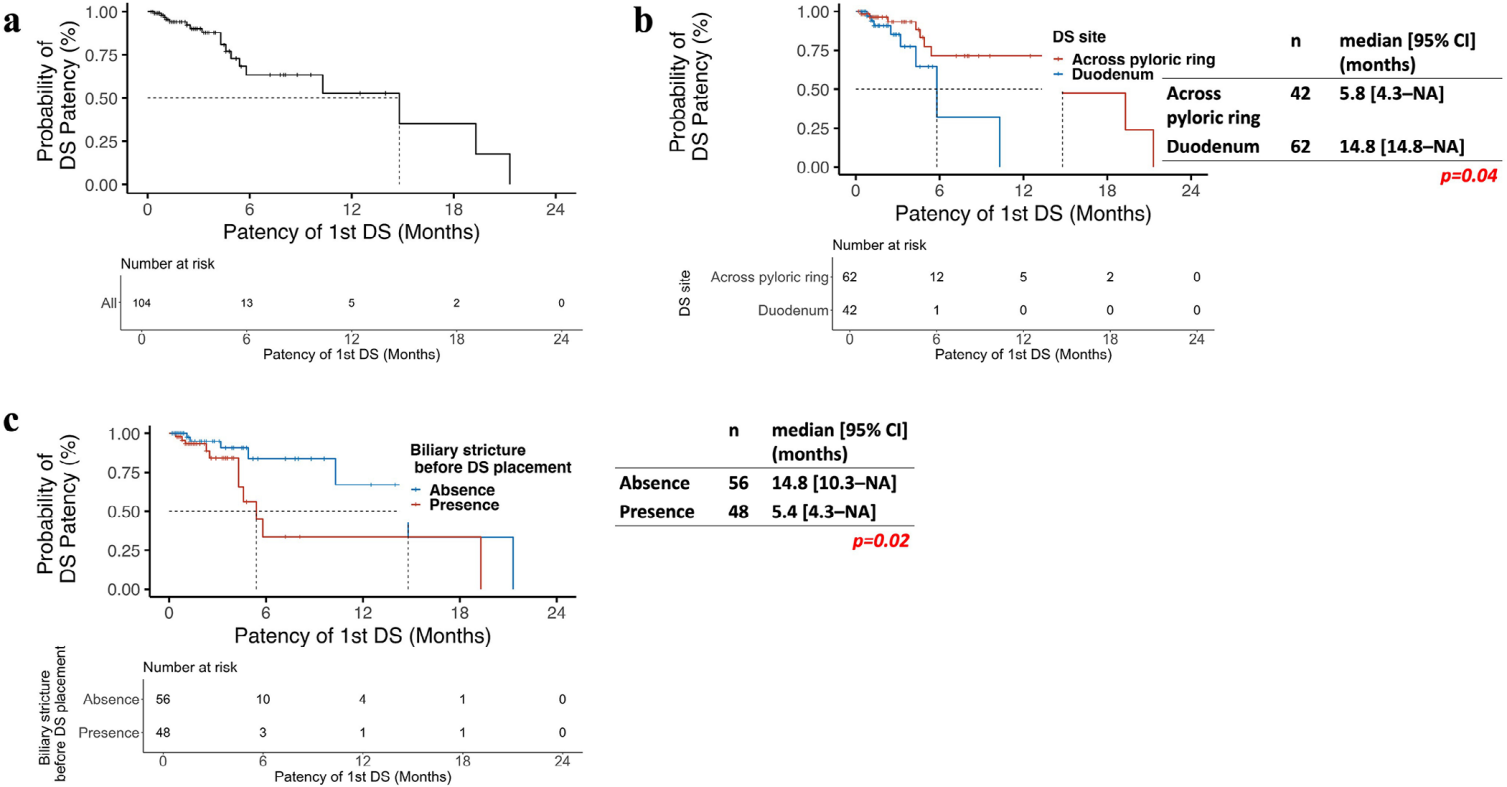
Duodenal stent patency: Kaplan–Meier curves showing duodenal stent patency: (a) all patients with overall clinical success; (b) comparison of stents placed across the pyloric ring versus within the duodenum; (c) patients with versus without pre‐existing biliary strictures. DS, duodenal stent.

Among the 104 patients who achieved overall clinical success, 18 (17.3%) experienced stent dysfunction. The causes included tumor ingrowth (*n* = 14), stent shortening (*n* = 4), poor stent expansion (*n* = 2), stent kinking (*n* = 1), stent migration (*n* = 1), and increased stricture length (*n* = 1) (Table 2). The median DS patency was 14.8 months (95% confidence interval [CI], 5.8–not available) (Figure 2). Multivariate analysis identified pre‐existing biliary stricture (hazard ratio [HR] 3.13, *p* = 0.03) and stent placement across the pylorus (HR 3.25, *p* = 0.033) as significant predictors of shorter stent patency (Table 3).

**TABLE 3:** Univariate and multivariate analyses for duodenal stent (DS) patency.

	Univariate			Multivariate		
Factors	HR	95% CI	*p*‐Value	HR	95% CI	*p*‐Value
Age at DS placement	1.02	0.96, 1.07	0.6			
Male	0.52	0.18, 1.49	0.2			
ECOG performance status, ≥2	1.09	0.34, 3.52	0.9			
Pancreatic cancer	0.76	0.24, 2.40	0.6			
Type I stenosis	2.12	0.74, 6.11	0.2			
Type II stenosis	1.47	0.54, 4.03	0.5			
Type III stenosis	0.34	0.11, 1.07	0.066			
Disease state, UR‐M/Recurrence	0.49	0.19, 1.32	0.2			
Ascites	0.66	0.08, 5.28	0.7			
Dissemination	1.02	0.34, 3.05	>0.9			
Biliary stricture before DS placement	3.06	1.11, 8.46	0.031	3.13	1.08, 9.10	0.03
DS length, ≥10cm	3.48	1.08, 11.2	0.037	2.77	0.86, 8.88	0.069
Across the pyloric ring placement	2.9	1.02, 8.24	0.045	3.25	1.10, 9.58	0.033
GOOSS score 0 before DS	1.95	0.73, 5.17	0.2			
Anti‐tumor treatment before DS	1.52	0.52, 4.40	0.4			
Anti‐tumor treatment after DS	0.84	0.19, 3.78	0.8			
Era, 2018–2024	2.52	0.57, 11.2	0.2			
GPS score 2	0.86	0.29, 2.52	0.8			
High NLR	1	0.36, 2.77	>0.9			
High CAR	0.85	0.29, 2.47	0.8			
High PLR	1.61	0.55, 4.65	0.4			
High LMR	0.48	0.17, 1.36	0.2			

Abbreviations: CAR, C‐reactive protein‐to‐albumin ratio; CI, confidence interval; DS, duodenal stent; GOOSS, gastric outlet obstruction scoring system; GPS, Glasgow prognostic score; HR, hazard ratio; LMR, lymphocyte‐to‐monocyte ratio; NLR, neutrophil‐to‐lymphocyte ratio; PLR, platelet‐to‐lymphocyte ratio.

**FIGURE 3:**
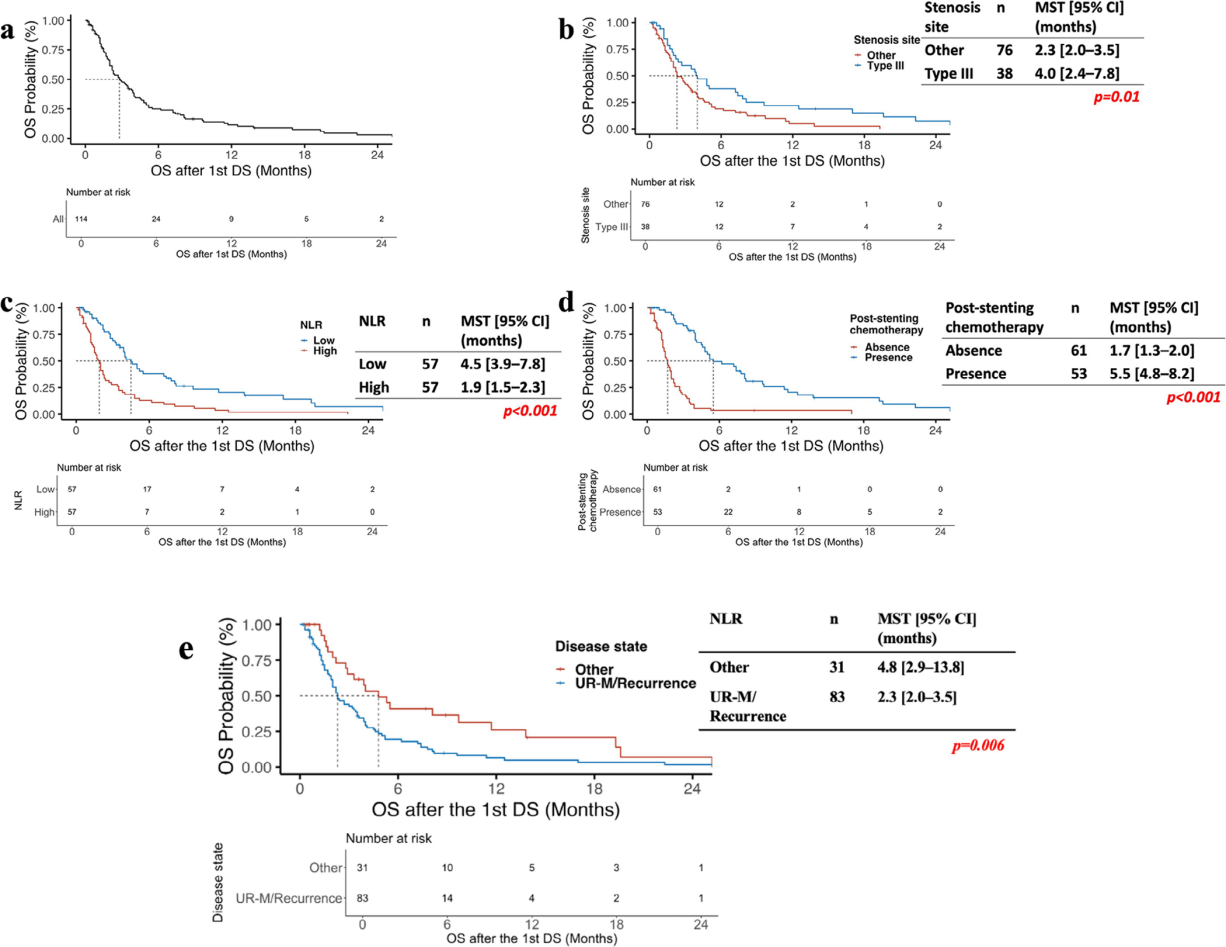
Kaplan–Meier survival curves following initially duodenal stent placement: (a) OS of all patients; (b) OS in patients with type III stenosis versus other types; (c) OS in patients with high versus low NLR; (d) OS in patients receiving versus not receiving post‐stenting anti‐tumor therapy; (e) OS in patients with disease state classified as UR‐M/Recurrence versus Other. OS, overall survival; DS, duodenal stent; NLR, neutrophil‐to‐lymphocyte ratio.

The median OS following DS placement was 2.8 months (95% CI, 2.2–3.9) (Figure 3). High NLR (HR 2.53, *p* < 0.001) and a disease state of UR‐M/Recurrence (HR 1.77, *p* = 0.022) were significantly associated with shorter OS, whereas type III stenosis (HR 0.51, *p* = 0.008) and post‐stenting antitumor therapy (HR 0.27, *p* < 0.001) were associated with prolonged survival (Table 4). In the PSM analysis (Supporting Information and Figure S1), OS remained significantly longer in patients receiving post‐stenting chemotherapy compared to those who did not (median OS: 5.0 vs. 2.0 months; log‐rank *p* < 0.001), supporting the robustness of the survival benefit observed.

**TABLE 4:** Univariate and multivariate analyses for overall survival following duodenal stent (DS) placement.

	Univariate			Multivariate		
Factors	HR	95% CI	*p*‐Value	HR	95% CI	*p*‐Value
Age at DS placement	1.01	0.99, 1.03	0.4			
Male	0.82	0.52, 1.28	0.4			
ECOG performance status, ≥2	2.07	1.36, 3.14	<0.001	1.39	0.85, 2.26	0.2
Pancreatic cancer	0.81	0.51, 1.30	0.4			
Type I stenosis	1.18	0.77, 1.80	0.4			
Type II stenosis	1.55	1.00, 2.42	0.052			
Type III stenosis	0.56	0.36, 0.90	0.015	0.51	0.30, 0.85	0.008
Disease state, UR‐M/Recurrence	1.95	1.20, 3.16	0.007	1.77	1.07, 2.95	0.022
Ascites	1.79	1.11, 2.90	0.017	1.54	0.91, 2.60	0.11
Dissemination	1.23	0.81, 1.87	0.3			
Biliary stricture before DS placement	1.49	0.98, 2.24	0.06			
DS length, ≥10cm	1.57	1.03, 2.40	0.036	1.31	0.84, 2.06	0.2
Across the pyloric ring placement	1.44	0.95, 2.19	0.09			
GOOSS score 0 before DS	1.18	0.79, 1.78	0.4			
Anti‐tumor treatment before DS	1.6	1.00, 2.56	0.052			
Anti‐tumor treatment after DS	0.2	0.13, 0.32	<0.001	0.27	0.15, 0.47	<0.001
Era, 2018–2024	0.93	0.61, 1.43	0.7			
GPS score 2	2.71	1.77, 4.15	<0.001	1.66	0.78, 3.51	0.2
High NLR	2.53	1.67, 3.83	<0.001	2.66	1.62, 4.34	<0.001
High CAR	2.15	1.42, 3.26	<0.001	0.87	0.41, 1.84	0.7
High PLR	1.76	1.17, 2.64	0.006	0.82	0.47, 1.41	0.5
High LMR	0.51	0.34, 0.77	0.002	0.95	0.54, 1.67	0.9

Abbreviations: CAR, C‐reactive protein‐to‐albumin ratio; CI, confidence interval; DS, duodenal stent; GOOSS, gastric outlet obstruction scoring system; GPS, Glasgow prognostic score; HR, hazard ratio; LMR, lymphocyte‐to‐monocyte ratio; NLR, neutrophil‐to‐lymphocyte ratio; PLR, platelet‐to‐lymphocyte ratio.

### 3.3 Procedure‐related AEs

**FIGURE 4:**
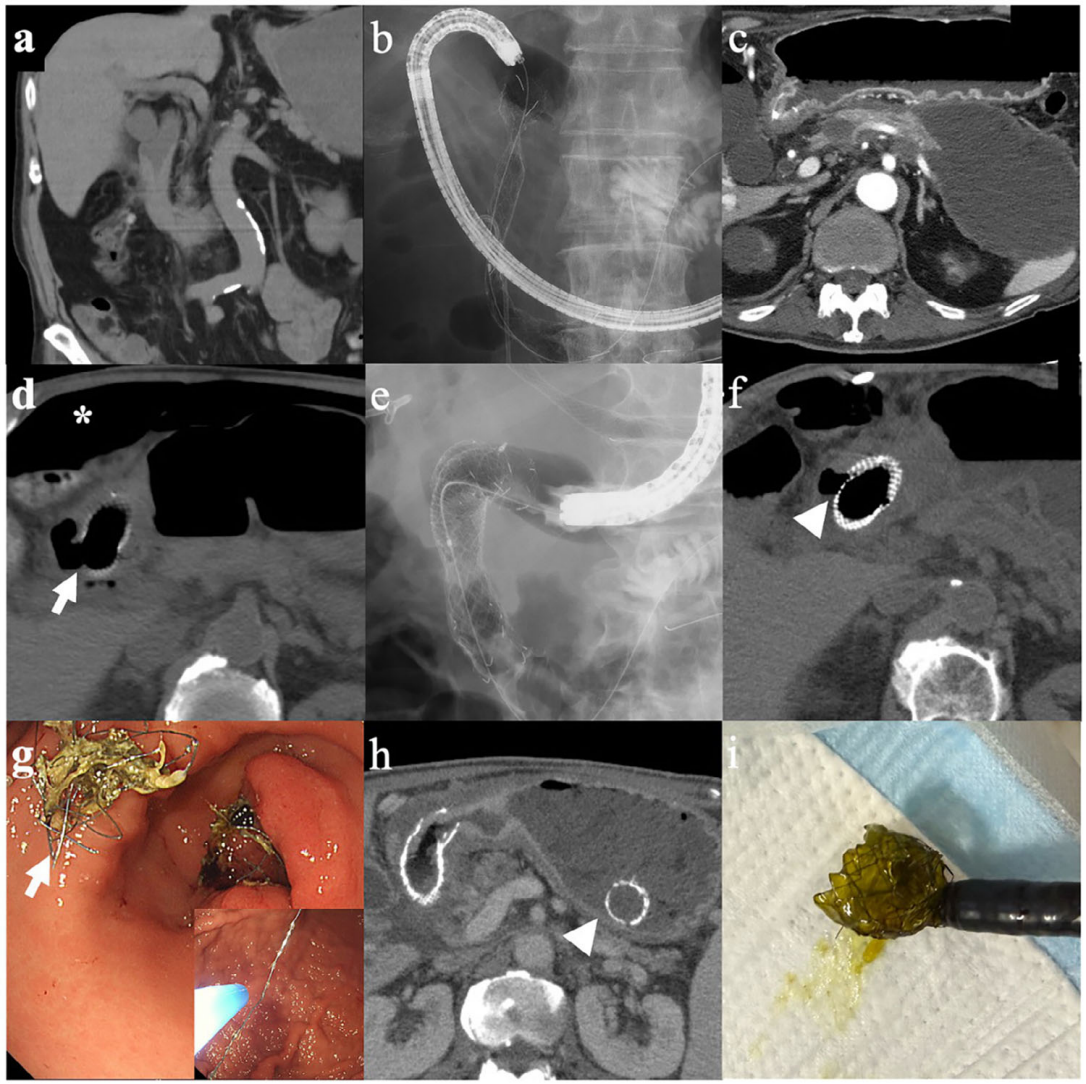
Representative cases of procedure‐related adverse events and stent dysfunction: (a–c) acute pancreatitis with walled‐off necrosis and obstructive jaundice following placement of an uncovered DS for type II stenosis secondary to ureteral cancer, treated with EUS‐HGS and EUS‐TD; (d–f) perforation after uncovered DS placement for type II stenosis caused by duodenal cancer managed with placement of a covered DS and percutaneous drainage (arrow: perforation site, *: free air, arrowhead: covered area); (g–i) recurrent GOO due to stent edge damage treated with APC trimming or DS replacement (arrow: stent edge trimmed with APC, arrowhead: fractured DS). DS, duodenal stent; EUS‐HGS, endoscopic ultrasound‐guided hepaticogastrostomy; EUS‐TD, EUS‐guided cystogastrostomy; GOO, gastric outlet obstruction; APC, argon plasma coagulation.

AEs following the initial DS placement were observed in 14 patients (12.3%), including jaundice/cholangitis (6.1%), pancreatitis (2.6%), and bleeding (2.6%). Isolated cases of perforation, Mallory‐Weiss syndrome, and stress cardiomyopathy were also reported (Table 2). One patient developed severe pancreatitis with walled‐off necrosis and concomitant obstructive jaundice attributed to DS placement. The condition was successfully managed using endoscopic ultrasound‐guided cyst drainage and hepaticogastrostomy; however, the patient experienced prolonged hospitalization and a decline in PS (Figure 4a–c). Another patient with perforation was treated by an additional covered stent placement along with percutaneous drainage (Figure 4d,e). However, one case involving tumor‐related bleeding resulted in mortality due to uncontrolled hemorrhage.

### 3.4 Recurrence of GOO and Reintervention With DS

**FIGURE 5:**
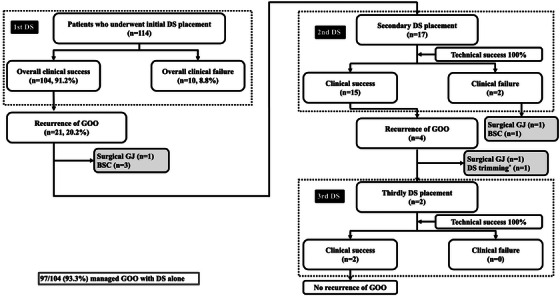
Clinical course of patients receiving second or subsequent stent placement: Although three patients eventually required surgical gastrojejunostomy, the majority (93.3%; 97/104) were managed with DS alone. DS, duodenal stent; GOO, gastric outlet obstruction.

Recurrent GOO occurred in 21 of the 104 patients (20.2%) who initially achieved clinical success (Figure 5). A cumulative total of 19 patients required second or third DS placement. All procedures were technically successful, with a clinical success rate of 89.5% and no reported complications (Table 5). The mean GOOSS improved from 1.26 ± 1.28 pre‐procedure to 2.74 ± 0.81 post‐procedure. The median stent patency was not reached during the observation period. Among patients who achieved clinical success, four (21.1%) developed stent dysfunction, including two cases of tumor ingrowth and two cases of stent breakage (Figure 5). Ultimately, only three patients required surgical GJ, whereas the majority (93.3%; 97/104) were managed with DS alone.

**TABLE 5:** Clinical outcomes of the second and third duodenal stent (DS).

	*n* = 19
Time to oral intake (days), median [range]	1 [1–2]
Technical success	19 (100)
Clinical success	17 (89.5)
GOOSS score before DS placement, mean ± SD	1.26 ± 1.28
GOOSS score after DS placement, mean ± SD	2.74 ± 0.81
Procedure‐related adverse events	0 (0)
Stent dysfunction	4 (21.1)
Tumor ingrowth	2 (10.5)
Stent fracture	2 (10.5)

Abbreviations: DS, duodenal stent; GOOSS, gastric outlet obstruction scoring system; SD, standard deviation.

## 4 Discussion

In this study spanning 15 years, we confirmed that DS placement is a highly effective and safe palliative intervention for MGOO. The technical and clinical success rates aligned with previous studies [1, 2], reaffirming the reliability of this approach. However, the 23.1% stent dysfunction rate highlights the need for careful patient selection and consideration of factors that may predispose to early stent failure.

Anatomical factors significantly influenced stent patency. Stents placed across the pyloric ring were associated with shorter patency, possibly due to increased mechanical stress from strong peristalsis and anatomical angulation, leading to stent dysfunction or migration. In such cases, flexible stents with a large proximal flare or alternative bypass procedures such as endoscopic ultrasound‐guided GJ (EUS‐GJ) may offer better durability. Pre‐existing biliary obstruction was also identified as an independent risk factor for stent dysfunction, consistent with previous articles [14]. Biliary stents may cause mechanical interference, while associated cholangitis or pancreatitis can lead to duodenal edema or anatomical changes impairing stent function. For such complex cases, multimodal approaches—including endoscopic ultrasound‐guided drainage—may be warranted. Their placement is also easier in tortuous anatomy. Uncovered SEMS (UCSEMS) have remained the standard at our institution, primarily due to their lower risk of migration, particularly in cases with extrinsic compression, sharp angulation, or tortuous anatomy. While covered SEMS (CSEMS) may reduce tumor ingrowth, they are associated with higher migration rates, as reported in previous studies [2, 15]. In our cohort, UCSEMS were predominantly used, with tumor ingrowth being the leading cause of stent dysfunction, consistent with Yamao et al. [15]. Although CSEMS may be considered in selected cases, UCSEMS offers a favorable balance between efficacy and safety in most clinical scenarios.

Notably, type III strictures were associated with longer OS, likely due to more localized disease and fewer mechanical complications. These findings support previous studies highlighting the prognostic relevance of the obstruction site [4]. Additionally, high NLR emerged as an independent predictor of poorer survival. This observation is consistent with prior research, including the study by Sugiura et al., who proposed a prognostic model incorporating NLR ≥4, liver metastasis, and cancer‐related pain to inform the choice between DS and surgical GJ [7]. Unlike PLR, CAR, or LMR, NLR may more accurately reflect the balance between tumor‐associated inflammation and immune suppression–both key in cancer progression and treatment response. Neutrophilia promotes tumor growth and metastasis via cytokine release and angiogenesis, while lymphopenia reflects impaired cell‐mediated immunity. This dual mechanism likely underlies the superior prognostic value of NLR, which has proven reliable across multiple cancer types, including gastrointestinal malignancies [6]. Our findings support incorporating such markers into routine clinical decision‐making to stratify patients. Post‐stenting chemotherapy significantly improved survival, emphasizing DS as a bridge to systemic therapy, aiding oral intake, nutritional support, and performance status—factors critical for tolerating chemotherapy. Although multivariable regression was used to adjust for confounding, residual bias could not be entirely excluded. Therefore, we performed a strict 1:1 PSM analysis, confirming the robust survival benefit of post‐stenting chemotherapy. These results emphasize the importance of coordinated multidisciplinary management. In practice, patients with high NLR may warrant early systemic therapy; those with biliary strictures or stents across the pylorus may benefit from alternative strategies like EUS‐GJ. Conversely, patients with type III stenosis and those eligible for post‐stenting chemotherapy may gain particular benefit from DS, supporting individualized treatment based on anatomical and inflammatory profiles.

Procedure‐related AEs occurred in 12.3% of patients, consistent with previously reported rates (5%–20%) [1, 2, 15]. In this study, cholangitis was the most common complication, especially in periampullary tumors, though unrelated to biliary stenting. Post‐procedural pancreatitis occurred exclusively in patients with preserved pancreatic parenchyma (i.e., non‐pancreatic malignancies), likely due to duct compression [16]. These findings underscore the importance of anatomical assessment and considering prophylactic drainage in high‐risk patients. Duodenal perforation, though uncommon, was successfully managed with CSEMS and adjunctive drainage, consistent with prior reports supporting its use in high‐risk or inoperable patients [17, 18]. Mechanical stent failures, including stent fracture and proximal dislocation, occurred mainly in mobile regions like the pylorus and were managed with APC trimming or additional stenting (Figure 4g–i), similar to prior reports in the antrum [19, 20]. In summary, procedure‐related AEs are influenced by tumor location, ductal anatomy, and stent positioning. Careful planning, including prophylactic drainage and stent selection, may help mitigate risks and optimize outcomes.

Our study also evaluated outcomes of reintervention. Among patients with recurrent GOO, secondary or tertiary stenting achieved an 89.5% clinical success rate without complications. So et al. demonstrated similar outcomes, identifying favorable PS and initial stent patency ≥6 months as predictors of success [21]. Sasaki et al. also reported high clinical success rates but highlighted an increased risk of perforation, particularly with WallFlex stents at duodenal bends, highlighting the need for anatomical consideration [22]. Despite a reintervention rate of approximately 20% [23], repeat stenting remains a viable, minimally invasive salvage option when appropriately planned and selected.

Another notable finding is the relatively stable clinical outcomes over the 15‐year study period, despite advancements in endoscopic and oncologic care. Various DS models were used, mostly UCSEMS. Although outcomes remained consistent, advances in flexibility, radial force, and delivery systems likely contributed to maintaining high success and low complication rates. Recent innovations—such as partially covered SEMS with over‐the‐scope clip fixation—have reduced stent migration and tumor ingrowth, key causes of dysfunction in MGOO [24]. Although our retrospective design limited model‐specific analysis, these refinements likely supported the favorable outcomes observed. This may reflect increasing patient complexity, underscoring the ongoing need for innovation in both procedural techniques and patient selection strategies. Recently, EUS‐GJ has emerged as a promising alternative, offering longer stent patency and fewer reinterventions than DS or surgical GJ [1]. However, its widespread adoption remains limited by technical complexity, required expertise, and lack of insurance coverage in Japan. Future research should prioritize comparative analyses, including cost‐effectiveness studies, to guide individualized treatment strategies and institutional decision‐making.

This study has limitations. First, its retrospective design may introduce selection and information biases, with data accuracy depending on medical records. Second, treatment decisions—including stent placement, stent type, and chemotherapy—were physician‐dependent, introducing variability. Third, as a single‐center study at a high‐volume institution, generalizability is limited. Additionally, tumor burden, nutritional status, and QOL data were not evaluated, and evolving treatment strategies over time may have influenced outcomes. However, this study, which identified anatomical and systemic prognostic factors associated with OS and stent patency after DS placement, is important for decision‐making in clinical practice. Prospective, multicenter studies with standardized protocols and patient‐reported outcomes are required to validate these findings.

## 5 Conclusion

DS placement remains a cornerstone in the palliative management of MGOO. Optimal outcomes require consideration of anatomical and systemic factors and integration of systemic therapies. Our findings offer practical insights into predictors of success following DS and support a personalized, multidisciplinary approach to improve patient selection, procedural strategy, and post‐procedural care.

Figure S1 Kaplan–Meier analysis of overall survival after duodenal stent placement in matched cohort stratified by post‐stenting chemotherapy. Kaplan–Meier survival curves comparing overall survival between patients with and without post‐stenting chemotherapy after propensity score matching. A significant survival difference was observed (*p* < 0.001).

## Conflicts of Interest

Nao Fujimori is an Associate Editor of DEN Open. The other authors declare no conflicts of interest.

## Ethics Statement

This study was approved by the Institutional Review Board of Kyushu University Hospital (23345–00) and conformed to the provisions of the Declaration of Helsinki.

## Consent

Informed consent was obtained in the form of opt‐out.

## Clinical Trial Registration

N/A

## Supporting information



deo270192‐sup‐0001‐FigureS1.jpg

**TABLE S1** Baseline characteristics of patients with and without post‐stenting chemotherapy after propensity score matching.

deo270192‐sup‐0003‐SuppMat.docx
