# Supplementary figures and images for: Efficacy and Safety of Duodenal Stenting for Malignant Gastric Outlet Obstruction: Insights From a 15‐year Single‐Center Experience
Source: DEN Open. 2025 Aug 25;6(1):e70192. doi: 10.1002/deo2.70192 (PMC12378013; doi:10.1002/deo2.70192)

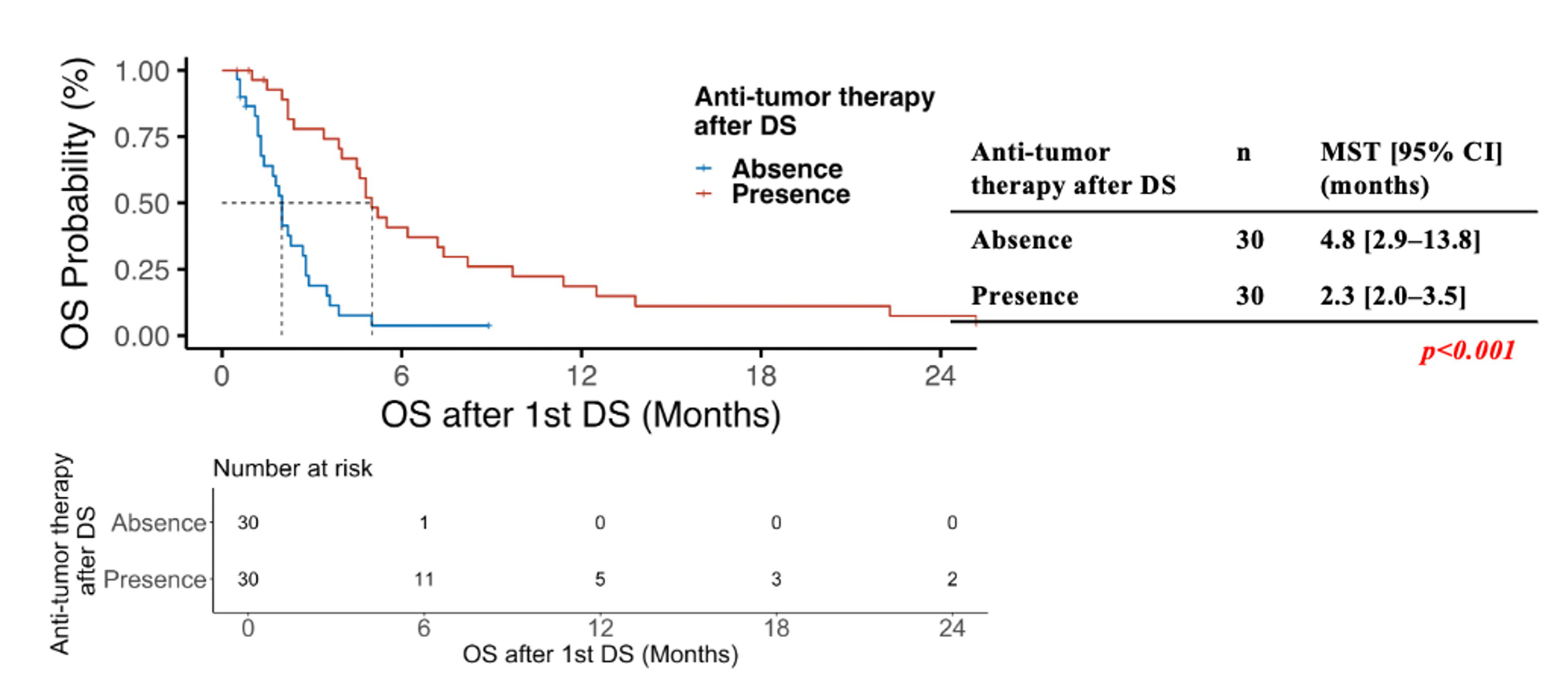

Supplement: Supplementary file 1 — deo270192‐sup‐0001‐FigureS1.jpg [file DEO2-6-e70192-s003.jpg]
