# Supplementary material for: Efficacy and Safety of Duodenal Stenting for Malignant Gastric Outlet Obstruction: Insights From a 15‐year Single‐Center Experience
Source: DEN Open. 2025 Aug 25;6(1):e70192. doi: 10.1002/deo2.70192 (PMC12378013; doi:10.1002/deo2.70192)
Supplement: Supplementary file 2 — TABLE S1 Baseline characteristics of patients with and without post‐stenting chemotherapy after propensity score matching. [file DEO2-6-e70192-s001.docx]

| Supplementary Table 1 Baseline characteristics of patients with and without post-stenting chemotherapy after propensity score matching. | | | | |
| --- | --- | --- | --- | --- |
|  | Post-stenting chemotherapy | |  |  |
| variable | Absence (n=30) | Presence (n=30) | p value | test SMD |
| Age at DS placement, mean (SD) | 68.89 (11.71) | 67.03 (7.41) | 0.464 | 0.19 |
| Male | 23 (76.7) | 23 (76.7) | 1.000 | <0.001 |
| ECOG performance status, ≥2 | 9 (30.0) | 9 (30.0) | 1.000 | <0.001 |
| Pancreatic cancer | 23 (76.7) | 23 (76.7) | 1.000 | <0.001 |
| Disease state, UR-M/Recurrence | 23 (76.7) | 22 (73.3) | 1.000 | 0.077 |
| Ascites, presence | 9 (30.0) | 6 (20.0) | 0.551 | 0.232 |
| Dissemination, presence | 12 (40.0) | 10 (33.3) | 0.789 | 0.139 |
| High NLR | 16 (53.3) | 17 (56.7) | 1.000 | 0.067 |
| SMD, standardized mean difference; DS, duodenal stent; SD, standard deviation; NLR, neutrophil-to-lymphocyte ratio. | | | | |
