# Supplementary material for: Efficacy and Safety of Duodenal Stenting for Malignant Gastric Outlet Obstruction: Insights From a 15‐year Single‐Center Experience
Source: DEN Open. 2025 Aug 25;6(1):e70192. doi: 10.1002/deo2.70192 (PMC12378013; doi:10.1002/deo2.70192)
Supplement: Supplementary file 3 — deo270192‐sup‐0003‐SuppMat.docx [file DEO2-6-e70192-s002.docx]

**Supplementary methods and results**

**Supplementary methods**

Stent device details

Stent selection was based on the site and length of the stricture. The following duodenal stents (DSs) were used at the discretion of the endoscopist:

- WallFlex DS: 6, 9, or 12 cm in length; 22 mm in body diameter (Boston Scientific, Marlborough, MA, USA)

- Niti-S DS: uncovered types (6, 8, 10, or 12 cm) and covered types (8 or 10 cm); all 22 mm in diameter (Taewoong Medical, Seoul, Korea)

- Evolution DS: 6, 9, or 12 cm; 22 mm in diameter (Cook Medical, Winston-Salem, NC, USA)

- JENTLLY NEO DS: 6, 8, 10, or 12 cm; 22 mm in diameter (Japan Lifeline Co., Ltd., Tokyo, Japan)

When multiple stents were placed in a single session, data from the longest stent were used for analysis.

Inflammation-based marker calculations

Systemic inflammation-based indices were calculated as follows:

- Neutrophil-to-lymphocyte ratio (NLR) = neutrophil count / lymphocyte count

- C-reactive protein-to-albumin ratio (CAR) = C-reactive protein / albumin

- Platelet-to-lymphocyte ratio (PLR) = platelet count / lymphocyte count

- Lymphocyte-to-monocyte ratio (LMR) = lymphocyte count / monocyte count

Each index was dichotomized into high and low groups based on the median value observed in the cohort.

Propensity score matching analysis

To minimize the impact of potential confounders between patients who received post-stenting chemotherapy and those who did not, a 1:1 propensity score matching (PSM) was performed. The propensity score was estimated using a logistic regression model that included the following clinically relevant covariates: age, sex, ECOG performance status, NLR, primary tumor site, disease state (unresectable/metastatic or recurrence), presence of ascites, and peritoneal dissemination.

Matching was conducted using a nearest-neighbor method without replacement, with a caliper width of 0.2 of the standard deviation of the logit of the propensity score. Covariate balance after matching was assessed using standardized mean differences (SMDs), with an SMD < 0.1 indicating adequate balance. All matched variables achieved acceptable balance post-matching.

**Supplementary results**

**Survival comparison after propensity score matching**

After matching, a total of 30 matched pairs were included in the survival analysis. The baseline characteristics of the matched cohort are shown in Supplementary Table 1. Kaplan–Meier survival analysis demonstrated a statistically significant improvement in overall survival in the chemotherapy group compared to the non-chemotherapy group (median OS: 5.0 vs. 2.0 months; log-rank P < 0.001) (Supplementary Fig. 1). This result supports the survival benefit of post-stenting chemotherapy even after adjusting for major clinical confounders.
